# Combining miRNA and mRNA Expression Profiles in Wilms Tumor Subtypes

**DOI:** 10.3390/ijms17040475

**Published:** 2016-03-30

**Authors:** Nicole Ludwig, Tamara V. Werner, Christina Backes, Patrick Trampert, Manfred Gessler, Andreas Keller, Hans-Peter Lenhof, Norbert Graf, Eckart Meese

**Affiliations:** 1 Department of Human Genetics, Saarland University, 66421 Homburg/Saar, Germany; Tamara.Werner@uniklinikum-saarland.de (T.V.W.); hgemee@uks.eu (E.M.); 2 Chair for Clinical Bioinformatics, Building E2.1, 66123 Saarbruecken, Germany; c.backes@mx.uni-saarland.de (C.B.); andreas.keller@ccb.uni-saarland.de (A.K.); 3 Center for Bioinformatics, Saarland University, Building E.1.1, 66041 Saarbruecken, Germany; ptrampert@bioinf.uni-sb.de (P.T.); lenhof@bioinf.uni-sb.de (H.-P.L.); 4 Developmental Biochemistry, Biocenter, and Comprehensive Cancer Center Mainfranken, University of Wuerzburg, 97074 Wuerzburg, Germany; gessler@biozentrum.uni-wuerzburg.de; 5 Department of Pediatric Oncology and Hematology, Medical School, Saarland University, 66421 Homburg, Germany; norbert.graf@uks.eu

**Keywords:** miRNA, Wilms tumor, blastemal, regressive

## Abstract

Wilms tumor (WT) is the most common childhood renal cancer. Recent findings of mutations in microRNA (miRNA) processing proteins suggest a pivotal role of miRNAs in WT genesis. We performed miRNA expression profiling of 36 WTs of different subtypes and four normal kidney tissues using microarrays. Additionally, we determined the gene expression profile of 28 of these tumors to identify potentially correlated target genes and affected pathways. We identified 85 miRNAs and 2107 messenger RNAs (mRNA) differentially expressed in blastemal WT, and 266 miRNAs and 1267 mRNAs differentially expressed in regressive subtype. The hierarchical clustering of the samples, using either the miRNA or mRNA profile, showed the clear separation of WT from normal kidney samples, but the miRNA pattern yielded better separation of WT subtypes. A correlation analysis of the deregulated miRNA and mRNAs identified 13,026 miRNA/mRNA pairs with inversely correlated expression, of which 2844 are potential interactions of miRNA and their predicted mRNA targets. We found significant upregulation of miRNAs-183, -301a/b and -335 for the blastemal subtype, and miRNAs-181b, -223 and -630 for the regressive subtype. We found marked deregulation of miRNAs regulating epithelial to mesenchymal transition, especially in the blastemal subtype, and miRNAs influencing chemosensitivity, especially in regressive subtypes. Further research is needed to assess the influence of preoperative chemotherapy and tumor infiltrating lymphocytes on the miRNA and mRNA patterns in WT.

## 1. Introduction

Wilms tumor (WT), or nephroblastoma, is the most common childhood renal tumor with an overall survival of more than 90% [1]. Nevertheless, 13% of all patients develop a relapse within the first two years after diagnosis [2]. In Europe, the International Society of Paediatric Oncology (SIOP) defined pre-operative chemotherapy as standard treatment, based solely on imaging studies [3,4]. Postoperative treatment depends on the local tumor stage and histological subtype according to the revised Stockholm classification [5]. The Children’s Oncology Group (COG) in North America also uses molecular markers for further risk stratified treatment, namely the loss of heterozygosity (LOH) for chromosomes 1p and 16q that is associated with an inferior outcome in a small subgroup of patients with WT [6,7,8,9]. The response to preoperative treatment plays an important role in SIOP. A remaining blastema after chemotherapy is a high risk factor and these patients need the same intensive treatment as patients with diffuse anaplasia [10].

MiRNAs are a class of small, endogenous, non-coding ribonucleic acids (RNAs) that are involved in the regulation of multiple physiologic processes, including apoptosis, proliferation, cell differentiation and are implicated in the pathogenesis of various diseases. The deregulation of miRNAs has been reported for many cancer types, including WT [11,12]. They can function as tumor suppressors and oncogenes at the same time [13,14,15]. Recent studies implicate that down- or upregulation of certain miRNAs may be the cause of non-responders to therapy or sensitizers to treatment, respectively [16,17]. In addition, circulating miRNAs have diagnostic and prognostic potential [18,19,20,21,22].

Recent evidence points toward a major role of miRNAs in WT genesis [23,24,25,26,27]. In a previous study we were able to determine treatment-independent miRNA signatures in the blood of patients with WT compared to controls [28]. There is also evidence for miRNAs expressed in WT tumors as markers for chemo-responsiveness in WT blastemas [29].

In the current study we assessed the tissue-based miRNA and gene expression profile of 36 WTs and four normal kidneys to identify miRNAs and genes differentially expressed in WT and in WT subtypes that might aid the development of a subtype specific miRNA biomarker.

## 2. Results

### 2.1. Deregulated miRNA Expression in Wilms Tumor (WT) Compared to Normal Tissue

**Figure 1:**
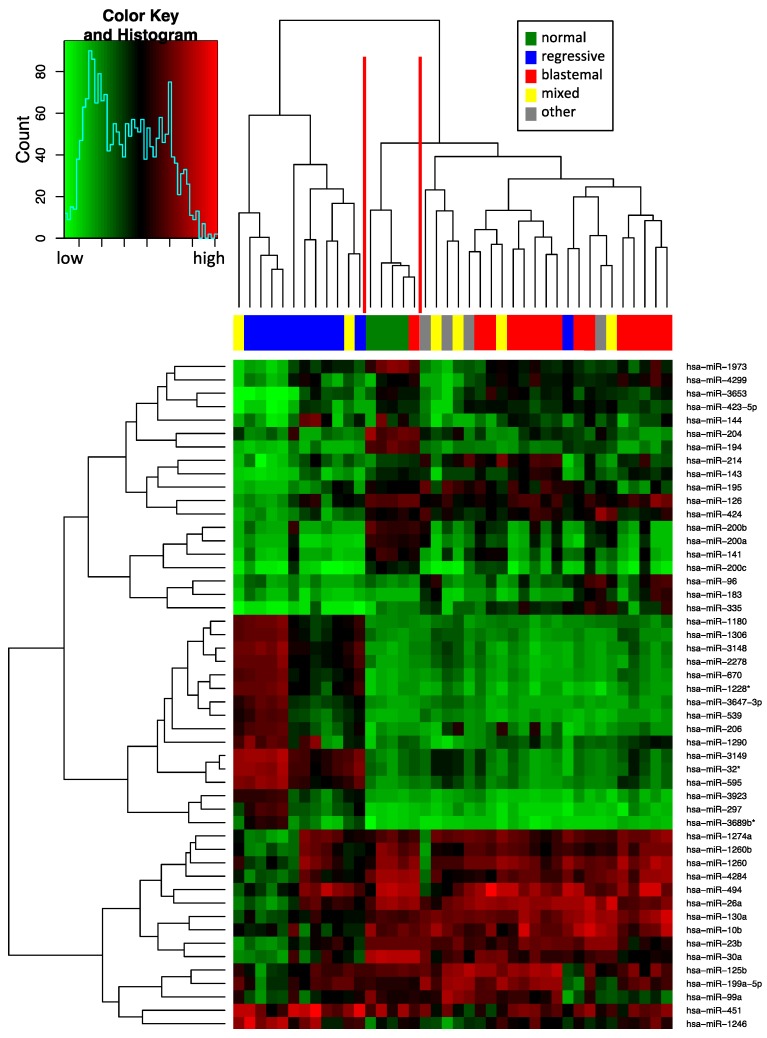
Unsupervised hierarchical clustering (Euclidian distance, complete linkage) of the 40 samples based on expression of the 50 with highest variance. The heatmap shows microRNAs with high expression in red, miRNAs with low expression in green. The red lines indicate three main clusters of samples.

We measured the expression of 1205 miRNAs by using Agilent microarrays based on miRBase version 16. Following background correction and quantile normalization, the miRNA expression profiles of 36 WTs and four normal kidney samples were used to identify miRNAs differentially expressed between the main WT subtypes, including regressive WT, mixed WT, and blastemal WT, and normal kidney samples. Unsupervised hierarchical clustering using the miRNAs with highest variance in our set revealed three main groups (Figure 1). One cluster contained 10 of the 11 regressive WT and two samples of mixed WT subtype, the second contained all four normal kidney samples and one blastemal WT, and the third contained the remaining 14 blastemal WTs, four mixed WTs, one regressive WT and four samples with other subtypes (stromal, epithelial, diffuse and focal anaplasia, one sample each). The one blastemal tumor sample that clusters together with the normal kidneys may have had a low percentage of tumor cells, therefore the miRNA profile might be masked by more abundant normal cells in the sample. In general, this analysis shows that tissue-borne miRNAs allow classification not only between normal kidneys and the WT, but also between regressive and blastemal WTs.

**Figure 2:**
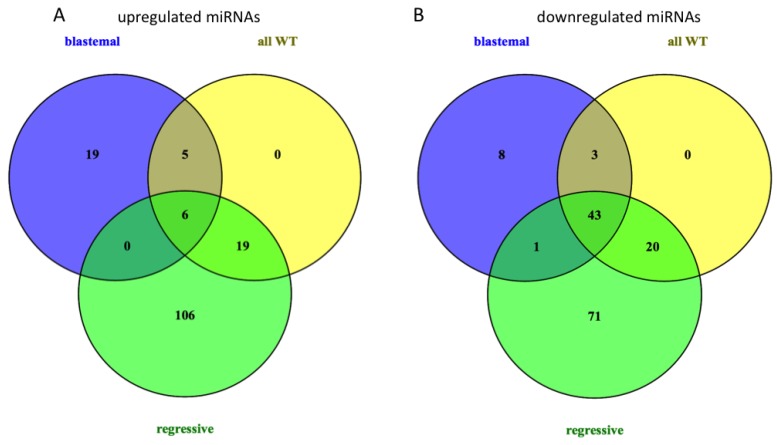
Venn diagram of (**A**) up- and (**B**) down-regulated miRNAs in Wilms tumors (WT) compared to normal kidneys.

Next, we performed unpaired *t*-tests to identify those miRNAs that show a differential abundance in all WTs compared to normal kidneys, and in blastemal and regressive subtypes, each also compared to a normal kidney. We considered those miRNAs as significantly differentially expressed that showed a fold change of >2 between WT samples and controls, and a *p*-value <0.05 in an unpaired *t*-test. In total, we identified 96 miRNAs that were significantly deregulated in all WT samples compared to controls (30 up in WT, 66 down in WT). An expression analysis of WT subtypes revealed 85 significantly deregulated miRNAs in the blastemal subtype (30 upregulated and 55 downregulated miRNAs) and 266 significantly deregulated miRNAs in the regressive subtype (131 upregulated and 135 downregulated miRNAs). The 15 most up- and downregulated miRNAs for each blastemal and regressive subtype, and for all WTs irrespective of subtype, are given in Table 1 (the full list of deregulated miRNAs is given in Supplemental Table S1). These data are largely in agreement with the clustering analysis. There is a different overall miRNA expression pattern in blastemal WTs as compared to regressive WTs, with blastemal WTs showing more downregulated miRNAs than the upregulated, while the regressive WTs show a more balanced number of up- and down-regulated miRNAs. The difference in miRNA expression in both subtypes also shows in the number of specifically expressed miRNAs, with 27 miRNAs deregulated solely in the blastemal subtype, and 177 miRNAs deregulated only in the regressive subtype as seen in Figure 2. There were also 49 miRNAs, which were found in all three comparisons, including three members of the miR-320 family, miR-1207-5p and miR-483-3p (upregulated) and 43 downregulated miRNAs, including several members of the miR-200 family, miR-141, and miR-429 (Figure 2).

### 2.2. Reverse Transcription Quantitative Realtime PCR (RT-qPCR) Validation of the miRNA Expression in WT Compared to Normal Tissue

For validation of the array data, we performed reverse transcription quantitative PCR (RT-qPCR) of five selected miRNAs in the same sample set that was used for array analysis (Table 2). These miRNAs were selected based on their differential expression in blastemal (miR-181b, -223, -320a, -485-3p) or regressive (miR-143) subtype in the array data and their previous association with either Wilms tumors or chemosensitivity. In detail, the miRNA miR-181b showed an upregulation with a fold change of 3.2 in a regressive WT *vs.* a normal kidney in the array analysis, and an upregulation with a fold change of 6.28 in the RT-qPCR analysis. Each of these analyses was statistically significant. The direction of deregulation of the miR-181b was also confirmed for the comparison between blastemal WT *vs.* a normal kidney: the array analysis showed an upregulation with a fold change of 1.2, and the RT-qPCR analysis showed a significant upregulation with a fold change of 3.52. Likewise, there was a confirmation of the upregulation of miR-223 in a regressive WT *vs.* a normal kidney with a significant fold change of 6.32 in the array analysis and of 60.34 in the RT-qPCR analysis. A significant upregulation was found for the miR-485-3p analysis, with a fold change of 3.97 in the array and a fold change of 11.11 in the RT-qPCR analysis in a regressive WT *vs.* a normal kidney. The upregulation of miRNA-320a in a blastemal WT *vs.* a normal kidney was also found both in the array and the RT-qPCR analysis, with significant fold changes of 2.69 and 2.08, respectively. MiR-143 was the only selected miRNA that showed a high and significant fold change in the array analysis, but not in the RT-qPCR analysis. Notably for miR-181b, miR-223, miR-485-3p and miR-320a, the direction of deregulation was also confirmed for the general comparison between WT and normal controls, with all four miRNAs upregulated both in the array and the RT-qPCR analysis.

### 2.3. Deregulated mRNA Expression in WT Compared to Normal Tissue

**Figure 3:**
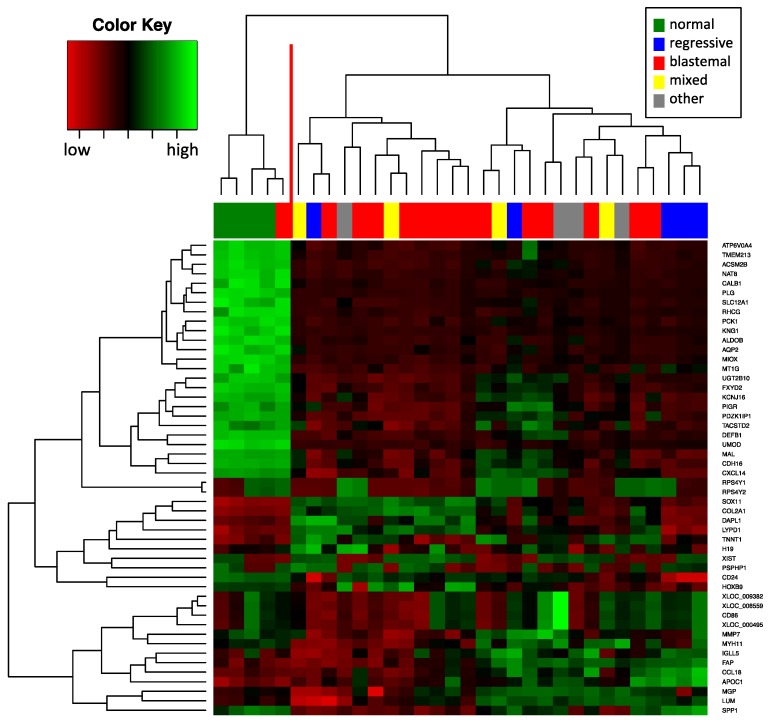
Unsupervised hierarchical clustering (Euclidian distance, complete linkage) of the 32 samples based on expression of the 50 mRNAs with highest variance. The heatmap shows mRNAs with high expression in red, mRNAs with low expression in green. The red line indicates two main clusters of samples.

We determined the mRNA expression profile using SurePrint G3 Human Gene Expression microarrays. In total, we analyzed the mRNA expression of 28 WTs and four normal kidney samples also used for the miRNA analysis. The WT samples included five regressive WTs, 15 blastemal WTs, four mixed WTs, one stromal WT, one epithelial WT, one diffuse anaplastic WT and one focal anaplastic WT. First, we used unsupervised hierarchical clustering depending on the expression of the 50 genes with highest variance to identify groups of tumors with similar gene expression patterns (Figure 3). One cluster contained all four normal kidney samples and the one blastemal tumor that clustered together with the normal kidneys in the miRNA analysis further supporting the hypothesis of low tumor cell content of the sample. As for the remaining groups, the clustering did not separate the different subgroups as clear as in the miRNA analysis. In general, mRNAs allowed classification only between a normal kidney and a WT, but not between regressive and blastemal WTs.

**Figure 4:**
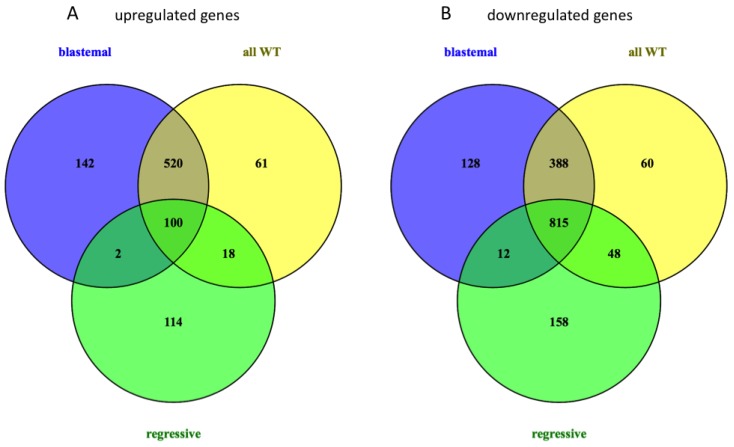
Venn diagram of (**A**) up- and (**B**) downregulated genes in Wilms tumor compared to normal kidneys.

For the identification of differentially expressed genes, we compared mRNA expression levels in blastemal, regressive and all WTs, combined with the gene expression level in normal kidneys using unpaired *t*-tests. Similar to the miRNA analysis, genes with a significant *p*-value in the *t*-test (*p* < 0.05) and a fold change of >2 were considered as significantly differentially expressed. A total of 2010 genes were significantly deregulated in all WTs *vs.* normal kidneys, including 1311 downregulated and 699 upregulated genes. With respect to subtypes, we found 2107 deregulated genes (764 up, 1343 down) in blastemal subtype *vs.* controls, and 1267 deregulated genes (234 up, 1033 down) in regressive subtype *vs.* controls. The Venn diagram in Figure 4 shows that 100 upregulated and 815 downregulated genes were common in the three comparisons. Subtype-specific deregulation was found for 256 genes (142 up, 114 down) in the blastemal and 286 genes (128 up, 158 down) in the regressive subtype. A list of all deregulated mRNAs is included in Supplemental Table S2. In general, we found an increased number of miRNAs and mRNAs each downregulated in WTs as compared to the normal tissues, most notably in blastemal WTs *vs.* normal kidneys.

### 2.4. Relation of mRNA and miRNA Expression

To further understand the relationship between miRNA and mRNA changes, and to specifically identify potentially relevant miRNA-mRNA target interactions, we computed the Pearson correlation coefficient for each miRNA/mRNA pair using the 96 deregulated miRNAs and 2010 deregulated mRNAs as input. In total, we found 13,026 miRNA-mRNA-pairs (1877 genes and 88 miRNAs) with inverse correlated expression (see Methods section and Supplemental Table S3). Of these potential interactions, 2844 are listed in miRWalk as predicted (854 genes and 70 miRNAs) and 36 thereof are listed in miRTarBase as validated (33 genes and 14 miRNAs). To identify the underlying pathways affected by potentially miRNA-regulated genes, we performed an overrepresentation analysis using all 1877 detected genes in GeneTrail [30]. The kyoto encyclopedia of genes and genomes (KEGG) pathways significantly enriched for deregulated genes are displayed in Table 3. We found three main groups of KEGG pathways: (i) metabolism: including multiple amino acid, drug and fatty acid metabolism pathways; (ii) signaling molecules and pathways: including “cell adhesion molecules” and Calcium- and PI3K-Akt signaling; and (iii) organismal systems pathways: including “Proximal tubule bicarbonate reclamation”, “Peroxisome proliferator-activated receptors (PPAR) signaling pathway” and “Endocrine and other factor-regulated calcium reabsorption”.

### 2.5. Chromosome Mapping of Deregulated miRNAs and mRNAs

To identify chromosome regions that are enriched for deregulated miRNAs, we performed a GeneTrail overrepresentation analysis (Table 4) [30]. We found significant enrichments of miRNAs downregulated in blastemal WTs *vs.* controls on chromosome 1 (11 precursors). MiRNAs that were downregulated in the regressive subtype were significantly enriched on chromosome X (31 precursors). Likewise, miRNAs that were downregulated in all WTs were enriched on both chromosome 1 and X. A significant enrichment of mRNAs downregulated in all WTs was found for mitochondrial DNA. There was no significant enrichment on any chromosomal location for upregulated genes or upregulated miRNAs.

## 3. Discussion

There is growing evidence that miRNAs play a major role in Wilms tumor genesis. Differential miRNA expression in WTs seems to be at least in part due to specific mutations that are mostly in the RNA binding domains of enzymes needed for the maturation of miRNAs, specifically drosha ribonuclease III (DROSHA), dicer 1 ribonuclease III (DICER1) and DGCR8 microprocessor complex subunit (DGCR8) [25,26,27,31,32]. Mutations in the DICER1 enzyme induce decreased abundance of mature 5p-miRNAs through the strand-specific inhibition of miRNA cleavage, whereas mutations in the DROSHA enzyme lead to a global decrease of canonical miRNAs in the mutated tumors [26,31]. Most interestingly, the DROSHA and DGCR8 mutations show a higher prevalence in WTs with blastemal histology [25,27].

In our current study, we found evidence for an overall altered miRNA expression pattern between different WT subtypes, most notably between regressive and blastemal WTs. The molecular difference between these two subtypes also shows in the number of deregulated miRNAs, which is three times higher in the regressive subtype as compared to the blastemal subtype (266 *vs.* 85). Likewise, the overall direction of deregulation is different between the two subtypes: approximately 2/3 of deregulated miRNAs downregulated in blastemal subtypes compared to normal kidneys in contrast to a similar number of up- and down-regulated miRNAs in the regressive subtype. These findings are in line with the abovementioned preferential loss of mature miRNA expression in WTs with blastemal histology due to the mutations in the miRNA processing enzymes. As for the biological explanation of the increased number of deregulated miRNAs in regressive compared to blastemal subtypes, and also for the more balanced ratio of up- and down-regulated miRNAs in regressive tumors, it is legitimate to speculate that these observations might be due to the increased presence of immune cells in this subtype.

Several specific miRNAs have already been associated with WTs [29,33,34,35]. Senanayake *et al.* [33] compared the expression of five miRNAs (miR-192, -194, -215, -200c, and -141), each between a normal kidney and WT of different histological subtypes. They found a significant downregulation of miR-192, miR-215 and miR-194 in all Wilms tumors irrespective of the subtype, and further a significant downregulation of miR-141 and miR-200c in blastemal and mixed WTs compared to normal kidneys [33]. Our findings support this result, as all of these miRNAs were significantly downregulated 21- to 46-fold in our WT set compared to a normal kidney. Kort *et al.* [34] demonstrated a significant overexpression of the oncomiR-1 cluster containing miR-17, -18a, -19b, -20a and -92 in WTs compared to normal kidneys, and an overexpression of several miRNAs, including miR-130b and miR-181b, in WTs compared to other renal tumors [34]. In our data, we found an overexpression of miR-18a and miR-130b in blastemal WTs and miR-181b in regressive WTs, each compared to normal kidneys. Furthermore, we could confirm the overexpression of miR-483-3p in WTs compared to normal kidneys. Previously, this overexpression was also shown by Veronese *et al.* [35]. There is also a considerable overlap of deregulated miRNAs in our study with the study of Watson *et al.* [29]. The results of Watson *et al.* [29] are, however, difficult to compare to our study. Watson *et al*. [29] compared miRNA expression in the blastemal component of high-risk patients with either the blastemal or the non-blastemal component of intermediate risk patients, and did not include normal kidney tissue as a reference. By contrast, our study focused on the differential expression of miRNA in blastemal (high-risk) and regressive (intermediate-risk) subtypes in comparison to normal kidneys, and without further information on the blastemal cell content of our probes. Of the 14 miRNAs reported to be upregulated in the high-risk blastemal compared to intermediate-risk non-blastemal component, we found that three miRNAs, namely miR-590-5p, miR-125a-5p and miR-19a, upregulated in blastemal WTs as compared to the regressive subtype. This analysis comes closest to the comparison of Watson *et al.* [29]. By contrast, out of the 17 reported miRNAs that were downregulated in high-risk blastemas, eight were also identified as deregulated in our study, with six miRNAs showing the opposite direction of deregulation, *i.e.*, an increased expression in blastemal WTs. Without data on the exact cellular composition of the regressive tumor samples used in our study, this obvious discrepancy could be attributed to possible residual blastemal cells in our regressive tumors.

In addition to the previously mentioned miRNAs, we found a strong downregulation of members of the miR-200 family and of the miR-30 family in both blastemal and regressive WTs. The downregulation of these miRNA families is associated with epithelial-to-mesenchymal transition, metastasis and chemoresistance in various cancers [36,37,38,39]. On the other hand, we found a marked upregulation of miR-320c/d/e in all subtypes and, additionally, miR-320a/b in blastemal subtypes. Regulating Wnt-signaling through its direct target beta-catenin, the overexpression of miR-320 has been shown to reverse epithelial-to-mesenchymal transition and inhibit stem cell-like properties in prostate cancer cells [40]. In addition, we detected a significant upregulation of miR-183, -301a/b and -335 exclusively in the blastemal subtype. The overexpression of miR-301b is known to promote invasion and contribute to chemoresistance in pancreatic carcinoma [41]. For the regressive subtype, we found significant downregulation of miR-195/497 and let-7a/c/d/e/f, and an upregulation of miRs-223 and -630. We confirmed the upregulation of miRs-181b, -223, and -320a in regressive WTs compared to normal tissue with RT-qPCR. These miRNAs are involved in modulating chemoresistance in various cancers [42,43,44,45,46,47]. For example, the overexpression of miR-223 has been shown to increase sensitivity to doxorubicin and paclitaxel in hepatocellular carcinoma cells [48], and sensitivity to cisplatin, mitomycin C and doxorubicin in esophageal adenocarcinoma cells [45]. Increased expression of miR-181b has been associated with increased chemosensitivity to vincristine and cisplatin in lung and gastric cancer cell lines [47], and temozolomide in glioblastoma cell lines [46]. To further judge the role of these miRNAs and their possible relevance to chemotherapy in blastemal *vs.* regressive WTs, it will be necessary to determine the cells of origin, *i.e.*, regressive tumor tissue or immune cells, and to link the abundance of these miRNAs to the clinical outcome in a prospective study.

Several chromosomal aberrations can be found in Wilms tumors, including losses on 1p, 4q, 7p, 9q, 11p, 11q, 14q, 16q, 21q and 22q, and gains on 1q, 3p, 4p, 7q, 8, 12 and 18 [49,50]. In our study, we performed an enrichment analysis for chromosomal regions enriched for deregulated miRNAs and genes. As for the chromosomal localization of miRNA precursor genes of deregulated miRNAs, we found a significant enrichment of downregulated miRNAs on chromosome 1 and chromosome X. The enrichment of downregulated miRNAs on chromosome 1 might be a consequence of the LOH 1p or the deletion of distinctive regions on 1p, including the loss of 1p36, which has been observed in about 10% of Wilms tumors patients [6,50]. In detail, we found downregulation of miRs-200a, -200b and -429, whose precursors are all located on 1p36.33. The deletion of regions on chromosome X have also been found in some cases, including Xp11.23 and Xq26.2-26.3 [50]. Downregulated miRNA with precursors in these regions include miR-363 and miR-424 for Xq26.2-26.3, and miR-362, -500a, -502, -532, and -660 for Xp11.23. Most interestingly, the latter are organized in a cluster in an intron of the gene CLCN5 that codes for a voltage-gated chloride ion channel expressed in renal proximal tubule cells in normal renal tissue. Therefore, downregulation of these miRNAs in Wilms tumors might be explained by the decreased expression of the ion channel in the tumor tissue. Notably, the CLCN5 gene is also among the commonly downregulated genes in our data set. In addition, our gene expression analysis revealed a significant enrichment of downregulated mRNAs on chromosome 4 and the mitochondrial genome. In total, nine of the 14 protein coding genes encoded on the mitochondrial genome were downregulated in WTs irrespective of subtype. Moderate to massive downregulation of mitochondrial mass has been shown for triphasic Wilms tumors, suggestive of a shift from oxidative phosphorylation activity towards anaerobic glycolysis in WTs [51]. This likely explains the observed downregulation of mitochondrial genes in WTs in our study. Deletions of distinctive regions or even the whole chromosome 4 have been reported in Wilms tumor patients [49].

Comparing the mRNA and the miRNA expression data, which are derived from the same tumor, we found that the overall direction of regulation does not show an inverse correlation between the miRNome and the transcriptome. By contrast, several of our comparisons show an overall deregulation of miRNAs and mRNAs in the same direction. For example, the majority of miRNAs (66 of 96) and the majority of mRNAs (1311 of 2010) were both downregulated in WTs as compared to normal kidney. Likewise, we found the majority of both miRNAs and mRNAs downregulated in the blastemal subtype as compared to the controls. These results suggest that the majority of miRNAs does not induce mRNA degradation. This question awaits further clarification by a simultaneous analysis of the proteome of the WTs and their subtypes. Besides these overall considerations, the availability of both miRNA and mRNA from the same origin allowed the search for potential miRNA-target gene interactions and pathways affected by these interactions. We identified 2844 potential interactions of 70 deregulated miRNAs, with 854 predicted gene targets with inverse correlation of miRNA and gene expression, including 36 already validated interactions. Among the predicted interactions was the interaction between the upregulated gene GLIPR1 and the downregulated miRNAs of the miR-30 family, *i.e.*, miR-30a/a*/b/c/c-2*/d/e/e*. The overexpression of GLIPR1 is known in Wilms tumors and occurs with a frequency of 67% [52]. Hypomethylation of the GLIPR1 promoter is even more common with 87% [52]. Downregulation of the miR-30 family in Wilms tumors might provide a further mechanism for Wilms tumors to increase GLIPR1 protein expression besides the increased transcription due to promoter hypomethylation. Additionally, we found an inverse correlation of miR-204 and the oncogenic transcription factor Meis homeobox 1 (MEIS1), which is consistent with a previous study showing a downregulation of miR-204 and an overexpression of MEIS1 in a high proportion of Wilms tumors [53]. This interaction has also been validated with reporter gene and protein assays [54]. It remains to be seen if the other predicted interactions are relevant for Wilms tumor genesis, or merely represent coincidental *in silico* associations. Here, we propose a number of possible miRNA-gene interactions that might play a role in Wilms tumors and that await further validation in an experimental setting.

An analysis of KEGG pathways enriched for genes of the interactions predicted by the overrepresentation analysis in Gene Trail identified three main groups of pathways, including metabolism, signaling molecules and pathways, and organism systems pathways. The individual pathways include pathways related to renal function, e.g., “Proximal tubule bicarbonate reclamation” and to know cancer-related pathways as for example PI3K-Akt signaling. The activation of PI3K-Akt signaling is a common feature in cancers, resulting in increased proliferation and reduced apoptosis. Since these pathway associations are derived from mRNA, not from protein data, and mRNA overexpressing genes are not necessarily overexpressed on protein level, e.g., due to their translational regulation by miRNAs, further analysis of the proteome will help to dissect the role of the deregulated miRNome in Wilms tumors.

## 4. Materials and Methods

### 4.1. Patient Samples

For identification of specific miRNA profiles in Wilms tumor (WT) tissue, we collected tumor tissue from 36 patients from the SIOP study including 11 regressive WTs, six mixed WTs, 15 blastemal WTs, and four other subtypes (stromal, epithelial, diffuse and focal anaplasia). As controls we used four samples of normal kidney tissue from patients with WTs. Written informed consent was obtained from all participants. Clinical details of the patients included in the analysis are provided in Supplemental Table S4. The research was approved by the local ethical committee (No. 136/01; 09/16/2010).

### 4.2. RNA Isolation

Total RNA including miRNAs was isolated from tumor and control tissues using the miRNeasy Kit (Qiagen, Hilden, Germany) according to manufacturer’s instructions. RNA concentration and integrity were assessed using NanoDrop2000 (Thermo Fisher Scientific, Waltham, MA, USA) and bioanalyzer run using the PicoRNA Chip (Agilent Technologies, Santa Clara, CA, USA).

### 4.3. miRNA Expression Profile

The miRNA expression profile of 36 Wilms tumors and four normal kidney controls was measured using the SurePrint G3 8x60k miRNA microarray (miRBase version 16, Cat. no. G4870A) containing probes for detection of 1205 mature human miRNAs and the miRNA Complete Labeling and Hyb Kit (Cat. No. 5190-0456) according to the manufacturers recommendations. Briefly, 100 ng total RNA including miRNA for each sample was dephosphorylated for 30 min at 37 °C using calf intestinal phophatase, denatured for 10 min at 100 °C using 100% dimethyl sulfoxide (DMSO) and subsequently labeled with Cy3-pCp for 2 h at 16 °C using T4 ligase. The labeled RNA for each sample was hybridized to the microarray for 20 h at 55 °C with 20 rpm rotation in the SureHyb chambers (Agilent). After two washing steps, the arrays were dried and scanned using the Agilent Microarray Scanner G2565BA (Agilent Technologies, Santa Clara, CA, USA) with 3 µm resolution in double-pass mode. Resulting tif-files were analyzed with the Agilent AGW Feature Extraction software (version 10.10.11, Agilent Technologies). Background corrected values were extracted, log-transformed and quantile normalized using freely available R software (v.2.14.2, www.r-project.org). We identified differentially expressed miRNAs in blastemal WTs, regressive WTs and all WT samples combined, each compared to the four normal kidney samples via an unpaired two-tailed *t*-test. We considered only miRNAs differentially expressed, that showed at least a 2-fold decreased or increased mean expression value and a *p*-value <0.05 in the *t*-test. P-values were adjusted using the Benjamini-Hochberg adjustment method [55]. Raw data has been deposited at GEO database (GSE57370). Unsupervised hierarchical clustering was performed with the R package heatmap.2 using complete linkage and euclidian distance applied to the normalized expression matrix using the 50 miRNA with highest variance in the data set. Venn diagrams were created using Venny 2.1.0 (http://bioinfogp.cnb.csic.es/tools/venny/index.html).

### 4.4. RT-qPCR Validation

For validation of miRNA array data, we performed quantitative real-time PCR of a total of five identified differentially regulated miRNAs (miRs-143, -181b, -223, -320a, and -485-3p) and RNU6B as control on a StepOnePlus Real-Time PCR system (Applied Biosystems, Foster City, CA, USA). The small RNA RNU6B is widely used as endogenous control in miRNA tissue studies by us and others [56,57,58,59]. In short, 100 ng total RNA of each sample (the same as used for array analysis) was used in first strand synthesis using the miScript II RT Kit (Qiagen) in a total volume of 10 µL using the high-spec buffer. The resulting complementary DNA (cDNA) was diluted 1:5 and 1 µL of the dilutions were used in RT-qPCR in a total volume of 10 µL using the miScript SYBR Green PCR Kit and the miScript Primer Assays (Qiagen) for each miRNA according to manufacturer’s recommendations. Reactions were performed in duplicates and mean threshold cycle (*C*_t_) values of duplicates were used to calculate Δ*C*_t_ for each miRNA compared to RNU6B in each sample. The mean Δ*C*_t_ of each considered group was calculated and significant expression differences were determined using two-tailed *t*-test (*p* < 0.05).

### 4.5. Gene Expression Profile

The mRNA expression profile of 28 Wilms tumors and four normal kidney controls was measured with SurePrint G3 Human Gene Expression 8x60Kv2 Microarray (Cat. no. G4851B, Agilent Technologies, Santa Clara, CA, USA) and the Low Input, one-color, Quick Amp Labeling Kit (Cat. no. 5190-2305, Agilent Technologies) according to the manufacturers recommendations. Briefly, 100 ng total RNA for each sample was reverse transcribed using Oligo-dT-T7 promotor primers for 2 h at 40 °C to obtain cDNA. Labeled cRNA was generated using Cy3-pCp and T7 RNA polymerase for 2 h at 40 °C and subsequently purified using RNeasy Mini kit (Qiagen) according to the protocol in the Low Input Quick Amp Labeling Kit, One Color, handbook. Purified cRNA was measured with the NanoDrop2000 Spectrophotometer (Thermo Fisher Scientific, Waltham, MA, USA) with the included Microarray Measurement protocol to determine output concentration and to ensure that labeled cRNA has sufficient quality for hybridization. 600 ng labeled cRNA were hybridized to the microarray slide for 17 h at 65 °C with 10 rpm rotation in the SureHyb chambers (Agilent). After washing and drying, the array was scanned in the Agilent microarray scanner G2565BA with 5 µm resolution. Resulting tif-files were analyzed with the Agilent AGW Feature Extraction software (version 10.10.11). Background corrected expression values of all 50,739 features were retrieved, log-transformed and quantile normalized. Next, we considered only features annotated with a *NCBI* gene symbol and calculated the median of replicated measurements of features displaying the same gene symbol resulting in a normalized expression matrix with 27,908 unique entries. The raw data has been deposited at GEO database (GSE66405). Unsupervised hierarchical clustering was performed with the R package heatmap.2 using complete linkage and euclidian distance applied to the normalized expression matrix using the 50 mRNAs with highest variance in the data set. Venn diagrams were created using Venny 2.1.0 (http://bioinfogp.cnb.csic.es/tools/venny/index.html).

### 4.6. miRNA-mRNA Correlation and Pathway Analysis

For the significantly deregulated 96 miRNAs and 2010 genes in WTs compared to normal tissue, we computed a Pearson correlation coefficient of expression for each mRNA-miRNA pair using R. For each potential interaction, defined as Pearson coefficient of the miRNA-mRNA pair <−0.5, we determined if the mRNA is a predicted or validated target of the miRNA using database searches in miRWalk (version 1.0) [60] and MirTarBase (release 4.5) [61] , respectively. Additionally, we performed an overrepresentation analysis of all negatively correlated genes using GeneTrail to analyze which KEGG pathways are significantly affected by gene expression deregulation in WTs [30].

## 5. Conclusions

Wilms Tumors exhibit differential miRNA and mRNA expression patterns, depending on their histological subtypes. It is, however, difficult to determine to what extent a pattern is attributed to cellular changes induced through chemotherapy or to the respective cell types. Towards a better understanding of the relationship between miRNA and mRNA expression in Wilms tumor subtypes, it is mandatory to analyze micro-dissected tumor areas with predominantly epithelial, stromal or blastemal cells prior to the administration of chemotherapy. Simultaneous analysis of the proteome will further clarify the downstream effects of the differential miRNA and mRNA expression patterns, including the effects on cellular pathways in the different cell types.

## Figures and Tables

**Table 1:** Deregulated miRNAs in Wilms tumors (WT) tissue compared to normal kidneys.

**15 Most Downregulated miRNAs in WT Subtypes Compared to Normal Kidneys**					
**Blastemal**		**Regressive**		**All WT**	
**miRNA**	**FC ^1^**	**miRNA**	**FC ^1^**	**miRNA**	**FC ^1^**
miR-194	48.95	miR-30a	143.28	miR-200a	46.47
miR-204	43.09	miR-194	64.67	miR-194	46.22
miR-200a	33.71	miR-200a	38.3	miR-204	36.81
miR-192	33.2	miR-204	36.5	miR-200b	35.38
miR-429	29.99	miR-30a *	34.58	miR-141	31.93
miR-215	25.52	miR-200b	31.58	miR-192	28.55
miR-200c	24.62	miR-192	26.64	miR-429	28.46
miR-200b	24.39	miR-1973	26.05	miR-215	24.02
miR-30a *	18.33	miR-141	24.1	miR-1973	22.35
miR-514	15.77	miR-4284	23.57	miR-200c	21.46
miR-141	14.47	miR-200c	21.44	miR-30a *	18.81
miR-1973	12.75	miR-429	20.79	miR-30a	16.95
miR-30a	11.46	miR-215	19.34	miR-514	9.56
miR-138	7.57	miR-30b	18.61	miR-30c-2 *	7.06
miR-30c-2 *	6.16	miR-26a	14.51	miR-30c	7.01
**15 Most Upregulated miRNAs in WT Subtypes Compared to Normal Kidneys**					
**Blastemal**		**Regressive**		**All WT**	
**miRNA**	**FC ^1^**	**miRNA**	**FC ^1^**	**miRNA**	**FC ^1^**
miR-199b-5p	7.87	miR-3149	25.05	miR-483-3p	8.11
miR-130b	6.16	miR-595	23.95	miR-34a	5.47
miR-335	5.50	miR-1290	23.61	miR-1207-5p	5.18
miR-483-3p	4.61	miR-32 *	20.63	miR-199b-5p	4.58
miR-183	4.48	miR-1225-5p	20.55	miR-1249	3.81
miR-301b	4.00	miR-3148	19.09	miR-130b	3.80
miR-301a	3.88	miR-1228 *	15.87	miR-1202	3.66
miR-34b *	3.79	miR-670	15.06	miR-320c	3.59
miR-34a	3.70	miR-4270	13.80	miR-1225-5p	3.50
miR-18a	3.58	miR-2278	13.23	miR-320d	3.34
miR-199a-3p	3.23	miR-1207-5p	13.21	miR-4270	3.25
miR-342-3p	3.08	miR-1306	13.07	miR-4281	3.01
miR-181c *	2.97	miR-4281	12.19	miR-320e	2.96
miR-1207-5p	2.95	miR-1246	11.66	miR-296-5p	2.67
miR-342-5p	2.82	miR-574-5p	10.91	miR-335	2.63

^1^ FC = Fold change; * indicates miRNA-star sequence.

**Table 2:** Reverse transcription quantitative realtime PCR (RT-qPCR) validation results.

miRNA	Fold Change		Direction of Regulation		Significance (*p*-Value)		Comparison
	Array	RT-qPCR	Array	RT-qPCR	Array	RT-qPCR	
miR-181b	1.21	3.52	up	up	ns ^1^	3.40 × 10^−4^	blastema *vs.* normal
	3.20	6.28	up	up	0.021	0.006	regressive *vs.* normal
	2.41	5.88	up	up	ns	7.45 × 10^−8^	all WT *vs.* normal
miR-223	1.74	1.99	up	up	ns	0.273	blastema *vs.* normal
	6.32	60.34	up	up	0.027	0.002	regressive *vs.* normal
	2.10	8.97	up	up	ns	0.008	all WT *vs.* normal
miR-320a	2.69	2.08	up	up	0.013	0.002	blastema *vs.* normal
	1.82	3.64	down	up	ns	0.026	regressive *vs.* normal
	1.11	2.85	up	up	ns	4.11 × 10^−5^	all WT *vs.* normal
miR-485-3p	1.02	2.20	up	up	ns	0.065	blastema *vs.* normal
	3.97	11.11	up	up	0.001	0.023	regressive *vs.* normal
	2.02	3.62	up	up	0.021	0.007	all WT *vs.* normal

^1^ ns = not significant.

**Table 3:** Kyoto encyclopedia of genes and genomes (KEGG) pathways enriched for potential target genes of deregulated miRNAs in WTs.

Group of Pathways	Individual Pathways
Metabolism	Tryptophan metabolism
	Starch and sucrose metabolism
	Retinol metabolism
	Phenylalanine metabolism
	Pentose and glucuronate interconversions
	Metabolism of xenobiotics by cytochrome P450
	Metabolic pathways
	Histidine metabolism
	Glycine, serine and threonine metabolism
	Fatty acid metabolism
	Fatty acid degradation
	Drug metabolism—other enzymes
	Drug metabolism—cytochrome P450
	Carbon metabolism
	Butanoate metabolism
	Ascorbate and aldarate metabolism
	Arginine and proline metabolism
	Arachidonic acid metabolism
	Alanine, aspartate and glutamate metabolism
Signal transduction and signaling molecules	Calcium signaling pathway
	Cell adhesion molecules (CAMs)
	Extracellular matrix (ECM)-receptor interaction
	Neuroactive ligand-receptor interaction
	PI3K-Akt signaling pathway
Cellular processes	Phagosome
	Cell cycle
	Focal adhesion
Organismal systems (immune, endocrine, digestive, excretory, sensory)	Renin-angiotensin system
	Proximal tubule bicarbonate reclamation
	Protein digestion and absorption
	PPAR signaling pathway
	Olfactory transduction
	Mineral absorption
	Gastric acid secretion
	Endocrine and other factor-regulated calcium reabsorption
	Complement and coagulation cascades
	Bile secretion
	Aldosterone-regulated sodium reabsorption
Human Diseases	Chemical carcinogenesis
	Amoebiasis

**Table 4:** Chromosomal enrichment analysis of miRNAs and genes downregulated in WT tissue.

**Chromosome (chr)**	**miRNA**								
	**Blastemal**			**Regressive**			**All WT**		
	**Expected**	**Observed**	***p***	**Expected**	**Observed**	***p***	**Expected**	**Observed**	***p***
Chr 1	3.97	11	0.01	–	–	–	5.09	12	0.02
Chr X	–	–	–	12.43	31	<0.001	5.76	18	<0.001
**Chromosome (chr)**	**Genes**								
	**Blastemal**			**Regressive**			**All WT**		
	**Expected**	**Observed**	***p***	**Expected**	**Observed**	***p***	**Expected**	**Observed**	***p***
Chr 4	40.55	61	0.01	–	–	–	40.71	64	0.002
Mitochondrial genome	0.71	11	<0.001	0.55	9	<0.001	0.71	9	<0.001

## Supplementary Materials

Supplementary materials can be found at http://www.mdpi.com/1422-0067/17/4/475/s1.

## Abbreviations

COG	Children’s Oncology Group
FC	fold change
LOH	loss of heterozygosity
RT-qPCR	reverse transcription quantitative PCR
SIOP	International Society of Paediatric Oncology
WT	Wilms tumor
